# Factors associated with improved walking in older people during hospital rehabilitation: secondary analysis of a randomized controlled trial

**DOI:** 10.1186/s12877-021-02016-0

**Published:** 2021-01-31

**Authors:** Catherine M Said, Jennifer L McGinley, Cassandra Szoeke, Barbara Workman, Keith D. Hill, Joanne E Wittwer, Michael Woodward, Danny Liew, Leonid Churilov, Julie Bernhardt, Meg E Morris

**Affiliations:** 1 Melbourne School of Health Sciences, The University of Melbourne, Parkville, Australia; 2 Physiotherapy Department, Western Health, St Albans, Australia; 3 Australian Institute for Musculoskeletal Science (AIMSS), Sunshine Hospital, St Albans, Australia; 4 Physiotherapy Department Austin Health, Heidelberg, Australia; 5 Healthy Ageing Program, Department of Medicine, The University of Melbourne, Centre for Medical Research, Parkville, Australia; 6 The Royal Melbourne Hospital, Parkville, Australia; 7 Institute for Health and Ageing, Australian Catholic University, Fitzroy, Australia; 8 Rehabilitation and Aged Care Services, Monash Health, Cheltenham, Australia; 9 Monash Ageing Research Centre (MONARC), Monash University, Cheltenham, Australia; 10 Rehabilitation, Ageing and Independent Living (RAIL) Research Centre, School of Primary and Allied Health Care, Monash University, Peninsula Campus, McMahons Road, Frankston, Australia; 11 La Trobe Centre for Exercise and Sports Medicine Research, School of Allied Health, La Trobe University, Bundoora, Australia; 12 Aged Care Services, Austin Health, Heidelberg, Australia; 13 Department of Medicine, The University of Melbourne, Parkville, Australia; 14 School of Public Health and Preventive Medicine, Monash University, Melbourne, Australia; 15 Department of Medicine (Austin Health) and Melbourne Brain Centre at Royal Melbourne Hospital, Melbourne Medical School, The University of Melbourne, Heidelberg, Australia; 16 Stroke Division, The Florey Institute of Neuroscience and Mental Health, Heidelberg, University of Melbourne, Heidelberg, Australia; 17 CRE Stroke Rehabilitation and Recovery, Heidelberg, Australia; 18 Victorian Rehabilitation Centre, Healthscope Australia, Melbourne, Australia

**Keywords:** Mobility limitation, Rehabilitation, Exercise therapy, Hospitalization, Aged, Aged, 80 and over

## Abstract

**Background:**

Older people are often admitted for rehabilitation to improve walking, yet not everyone improves. The aim of this study was to determine key factors associated with a positive response to hospital-based rehabilitation in older people.

**Methods:**

This was a secondary data analysis from a multisite randomized controlled trial. Older people (n= 198, median age 80.9 years, IQR 76.6- 87.2) who were admitted to geriatric rehabilitation wards with a goal to improve walking were recruited. Participants were randomized to receive additional daily physical therapy focused on mobility (n = 99), or additional social activities (n = 99). Self-selected gait speed was measured on admission and discharge. Four participants withdrew. People who changed gait speed ≥0.1 m/s were classified as ‘responders’ (n = 130); those that changed <0.1m/s were classified as ‘non-responders’ (n = 64). Multivariable logistic regression explored the association of six pre-selected participant factors (age, baseline ambulation status, frailty, co-morbidities, cognition, depression) and two therapy factors (daily supervised upright activity time, rehabilitation days) and response.

**Results:**

Responding to rehabilitation was associated with the number of days in rehabilitation (OR 1.04; 95% CI 1.00 to 1.08; p = .039) and higher Mini Mental State Examination scores (OR 1.07, 95% CI 1.00 – 1.14; p = .048). No other factors were found to have association with responding to rehabilitation.

**Conclusion:**

In older people with complex health problems or multi-morbidities, better cognition and a longer stay in rehabilitation were associated with a positive improvement in walking speed. Further research to explore who best responds to hospital-based rehabilitation and what interventions improve rehabilitation outcomes is warranted.

**Trial registration:**

Australian New Zealand Clinical Trials Registry ACTRN12613000884707; ClinicalTrials.gov Identifier NCT01910740.

## Background

One of the most frequent reasons older people are admitted for hospital-based rehabilitation is to improve their walking. While physical rehabilitation programs lead to improved outcomes for many people, [1, 2] not everyone improves [3]. One of the challenges is identifying who is likely to respond to specific physical therapy interventions. Understanding factors associated with positive responses to rehabilitation could assist health professionals develop evidence-based, rehabilitation programs and ensure efficient use of finite resources.

An older person’s response to rehabilitation whilst in hospital may be influenced by their clinical status. Older people are more likely to have multiple co-morbidities, cognitive impairment and frailty [4, 5]. These factors are associated with less optimal health outcomes, [6, 7] although associations with response to rehabilitation is less well understood. Some studies have shown that co-morbidities, cognition and frailty are associated with poor rehabilitation outcomes; [8–10] others have found no association with rehabilitation response [11–18]. Factors such as age, ambulation status on admission to hospital rehabilitation and depression may also affect rehabilitation outcomes [10, 19].

Characteristics of the therapy intervention may also impact an older person’s response to rehabilitation. The link between exercise dosage and outcomes is well established for people with stroke, [20–22]. Parkinson’s disease [2] and for fall prevention in older people [23]. The link between physical activity and outcomes is less clear for older people undergoing hospital-based rehabilitation, who often have complex health care needs.

The aim of this investigation was to understand key factors associated with a positive response to hospital-based rehabilitation in older people, as indicated by a clinically meaningful increase in gait speed. Our recent multi-site randomized controlled trial (RCT) conducted in older people receiving multidisciplinary, hospital-based rehabilitation, found additional supervised activity focussed on upright tasks such as standing and walking did not lead to improved gait speed [24]. The amount of time people spent performing upright physical activity during ‘usual care’ physical therapy sessions and the number of days over which the intervention was delivered were documented. Secondary analysis of data shall allow us to explore whether an older person’s response to rehabilitation was associated with their baseline personal characteristics or therapy characteristics. It was hypothesised that age, non-ambulant status at baseline, cognition, frailty, co-morbidities, and depression would reduce the likelihood of a positive response to hospital-based rehabilitation. Therapy factors including daily supervised upright activity time (in minutes) and the number of days in rehabilitation were predicted to be associated with a positive response.

## Methods

### Design

The full protocol has been published as have results of the RCT [24, 25]. The study was an investigator and assessor-blinded, parallel group multi-site RCT comparing outcomes following provision of a program of ‘enhanced physical activity’ or ‘usual care plus matched face to face contact time’ to 198 older people receiving hospital-based geriatric rehabilitation in the metropolitan area of Melbourne, Australia. Ethical approval was obtained from the relevant ethics committees in Australia prior to commencement. The trial was monitored by a management committee and an independent data safety monitoring committee.

### Participants

Participants were recruited from four participating geriatric rehabilitation wards at two Australian hospitals. These wards typically admit people who are medically stable but require multidisciplinary management or rehabilitation to maximise function. Eligible patients were aged over 60 years old with a goal to ‘improve mobility or walking,’ determined by admission referral or treating therapist. Participants were excluded if (1) there were medical restrictions limiting mobilisation, (2) goals were non-weight bearing (e.g. to improve slide-board transfers), (3) they were enrolled in another RCT, or (4) primary reason for admission was carer training or residential care placement. Informed consent was obtained from the participant or ‘responsible person’ within 48 hours of admission, with interpreters utilised as necessary.

After baseline data collection, participants were individually randomized to an intervention (enhanced physical activity; n = 99) or control group (usual care plus; n = 99) according to a computer-generated randomization procedure, stratified according to site and baseline ambulation status and performed by a third party. Both groups received usual care provided by a multidisciplinary team throughout their inpatient rehabilitation. Those in the *intervention arm* received a program to increase time participants spent performing upright activities, such as standing and walking. This was delivered daily (including weekends) throughout the inpatient stay and supervised by a physical therapist or assistant. Further details have been previously published [24]. Participants in the *control arm* were provided with additional social activities with minimal impact on mobility; including card or board games, conversation, reading or upper limb exercises. This was designed to match the additional face to face time provided to the intervention arm. Time spent performing various activities was recorded for all usual care physical therapy sessions and all intervention sessions.

### Assessments

Assessments were completed at baseline and discharge by an assessor blinded to group assignment. Baseline assessments were completed within 48 hours of admission. Discharge assessments were completed in hospital within 48 hours of discharge. For participants discharged to residential care, the date of completion of residential care paperwork was used in lieu of actual discharge date.

### Outcome measures

Full details on all outcome measures have been previously published; [25] those relevant to this analysis are summarized below.

#### Response to rehabilitation

The dependent variable was response to rehabilitation, determined by change in self-selected gait speed between admission and discharge. Gait speed was selected as it is clinically important, valid and responsive to change, [26–29] with a change ≥ 0.1 m/s indicating a substantial meaningful change [27]. Gait speed was assessed by the 6-meter walk test [30] with usual indoor gait aid, and participants unable to complete the test were given a score of 0 m/sec. Discharge data were missing for 19 participants. Four participants withdrew prior to this assessment and were unable to be included in this analysis. Blinded adjudication of the medical records was undertaken for the remaining 15 participants to determine whether the person was capable of completing the 6-meter walk test at discharge. Those able to complete the test had their baseline assessment carried forward; those unable to walk were assigned a score of 0 m/sec. Participants who increased gait speed ≥ 0.1 m/s between admission and discharge were classified as ‘responders’; participants who improved < 0.1 m/s were classified as ‘non-responders’.

#### Patient and therapy characteristics

Data were collected on eight variables hypothesised to be associated with response to rehabilitation. Patient factors were collected at baseline and included age, baseline ambulation status *(ambulant;* defined as able to walk with assistance of one person or independently or *non-ambulant;* defined as unable to walk or requiring assistance of two people), cognition [Mini Mental State Examination (MMSE)] [31], frailty (modified Fried Frailty Index), [32, 33] co-morbidities [Charlson Co-morbidity Index [34] (CCI)] and depression [Geriatric Depression Scale (GDS)] [35]. Therapy factors were collected during the hospital stay and included daily supervised upright activity, and days of rehabilitation. Daily supervised upright activity was calculated by combining time spent performing upright physical activity tasks (e.g. balance, walking, lower limb strengthening in standing) during usual care and intervention physical therapy sessions and calculating the median time in minutes for each participant. Days of rehabilitation was calculated as number of days between randomisation and discharge assessment.

### Sample size

Sample size was estimated for the primary hypothesis of the RCT and has been previously published [25]. No separate power analysis was conducted for this secondary analysis.

### Statistical analysis

In this secondary analysis, trial participants were analysed as a whole group. As daily upright activity time differed between intervention and control arms, group was not included in the analysis. Patient and care-related characteristics (age, baseline ambulation status, cognition, frailty, co-morbidities, depression, daily supervised upright activity time, and days in rehabilitation) were summarized as counts (proportions), means (SDs) or medians (IQRs) as appropriate. The univariate associations of these characteristics and the responder status was investigated using logistic regression modelling with corresponding effects reported as Odds Ratios (ORs) with 95 % confidence intervals (95 %CIs). The diagnostic utility of these characteristics for classifying the “responder” status was investigated using Receiver Operating Characteristics (ROC) analysis. Adjusted association between patient and care characteristics and the responder status was investigated using multivariable logistic regression with corresponding effects reported as adjusted Odds Ratios (aORs) with 95 %CIs.

All analyses were conducted using Stata 15IC statistical software (StataCorp, College Station, TX, USA); p-values < 0.05 were regarded as indicative of statistical significance. No correction for multiplicity of testing was undertaken due to the exploratory nature of the study.

## Results

Of the 194 participants included in analysis, 130 (67 %) participants were classified as responders and 64 (33 %) participants were classified as non-responders (four participants who withdrew could not be classified). Table 1 provides a description of the characteristics of each responder group. Responders had more days in rehabilitation than non-responders, but groups were similar on remaining variables.

**Table 1:** Characteristics of ‘responders’ and ‘non-responders’ to hospital rehabilitation^a^

	Responders (*n *= 130)	Non-responders (*n* = 64)
Age (years)	80.5 (75.4, 86.9)	82.7 (78.0, 88.7)
Male (n ,%)	54 (42 %)	29 (45 %)
Baseline ambulation status [Ambulant/Non-ambulant (n, %)]	95:35 (73 %:27 %)	51:13 (80 %:20 %)
Mini Mental State Examination	25 (21, 27) (*n* = 126)	24 (18, 28) (*n* = 59)
Frailty Index	2 (2, 3) (*n* = 126)	2 (2, 3) (*n* = 59)
Charlson Co-morbidity Index	2 (1, 3)	2 (1, 3)
Geriatric Depression Scale	4 (3, 7) (*n* = 125)	5 (3, 7) (*n* = 60)
Daily supervised upright activity time (mins)	25 (15, 35)	21 (10, 35)
Days in rehabilitation	16 (12, 26)	14 (8, 22)

^a^ reported as median (IQR) unless otherwise stated

Results of the univariate analysis are presented in Table 2. There were few participants with Fried frailty scores of 0 (*n* = 2), 4 (*n* = 12) and 5 (*n* = 1). Frailty was therefore dichotomised and participants were reclassified as *not frail* (scores 0–2) or *frail* (scores 3–5). The number of days in rehabilitation was the only variable associated with increased odds of a person having a positive response to rehabilitation.

**Table 2:** Univariate regression of factors influencing response to rehabilitation

	Odds ratio	95 % CI	*p* value	ROC area
Age (per year)	0.97	0.93, 1.00	0.092	0.58
Baseline ambulation status (reference *non-ambulant*)	1.45	0.70, 2.97	0.317	0.53
Fried Frailty Status (reference *not frail*) ^a^	1.48	0.79, 2.79	0.266^a^	0.55
Charlson Co-morbidity Index (per one point increase)	1.00	0.88, 1.14	0.959	0.50
Mini Mental State Examination (per one point increase)	1.04	0.98, 1.10	0.157	0.54
Geriatric Depression Scale (per one point increase)	1.00	0.91, 1.10	0.979	0.52
Daily supervised upright activity time (per min)	1.01	0.99, 1.03	0.414	0.54
Days in rehabilitation (per extra day)	1.03	1.00, 1.06	0.034	0.61

^a^*not frail* = scores 0, 1, 2

A multivariable model was constructed, which initially included all variables. This model demonstrated excessive collinearity; age was removed from the model, as other variables allowed more precise description of health status, and co-morbidities were removed as this included dementia which was captured with the MMSE. The resulting model included baseline ambulation status, frailty, cognition, depression, daily supervised upright activity time, and days in rehabilitation. As there was still evidence of collinearity, a model was developed with the removal of depression. The results of these two models were similar, thus the model which excludes depression has been reported. Higher MMSE scores (aOR 1.07; 95 % CI 1.00, 1.14, *p* = 0.048) and more days receiving rehabilitation (aOR 1.04 per day; 95 % CI 1.00–1.08, *p* = 0.039) were associated with increased odds of responding to rehabilitation. No association was found for baseline ambulation status, frailty and daily supervised upright activity time and the odds of responding to rehabilitation (see Table 3).

**Table 3:** Multivariable logistic regression for factors influencing response to rehabilitation

	Adjusted Odds ratio	95 % CI	*p* value
Baseline ambulation status (reference *nonambulant*)	2.15	0.81, 5.79	0.126
Fried Frailty Status (reference *not frail*)^a^	1.15	0.58, 2.28	0.58
Mini Mental State Examination (per one point increase)	1.07	1.00, 1.14	0.048
Daily supervised upright activity time (per min)	1.01	0.99, 1.04	0.311
Days in rehabilitation (per extra day)	1.04	1.00, 1.08	0.039

^a^*not frail* = scores 0, 1, 2

## Discussion

In hospitalized older people with muti-morbidity, only two thirds of participants achieved a ‘substantial meaningful’ improvement of 0.1 m/s in gait speed during multidisciplinary rehabilitation. Higher scores on the MMSE were associated with increased odds of responding to rehabilitation. Other participant characteristics including baseline ambulation status, frailty, co-morbidities, and depression were not related to improvements such as increased gait speed.

For admission to subacute rehabilitation the people in this trial needed to have the capacity to make meaningful gains and the study specifically recruited older people with a goal to improve walking or mobility. Despite our sample being biased towards responders, around one-third did not achieve a meaningful improvement in gait speed. Other studies of hospital rehabilitation have found as few as 44 % of people achieve a clinically meaningful response to rehabilitation [3]. Ability to respond to rehabilitation may be compromised in some older people due to new acute health issues or exacerbation of chronic medical issues; 5 % of our cohort did not complete their planned rehabilitation as they either died or were readmitted to the acute hospital.

The appropriateness of criteria for determining a clinically meaningful response to hospital-based rehabilitation may also require consideration. Use of the more modest indicator of a small meaningful change of 0.05 m/s, [27] would have resulted in classification of 75 % of our group as responders. We selected gait speed as our indicator of response as our study focussed on improving walking, however other indicators of rehabilitation success, such as Functional Independence Measure, may have led to different findings. A large proportion of non-responders within a group may also mask potential effectiveness of interventions. This may have contributed to the null finding in our primary analysis [24]. Further exploration of factors which differentiate responders and non-responders will help interventions to be directed and evaluated in specific cohorts.

We found better baseline cognition, assessed using MMSE, was associated with a slight increase in the odds of responding to rehabilitation, when other factors were taken into account. There is conflicting evidence in the literature about the impact of cognition on change in function during rehabilitation [10, 15, 17, 36]. Some studies have shown better cognition was associated with greater change in Barthel Score [17] and functional gain and relative functional gain during rehabilitation, measured using the Functional Independence Measure (FIM) [10, 36]. However, Muir- Hunter et al. [15] found that while a diagnosis of dementia impacted on the relative functional gain and relative functional efficiency in FIM total scores, dementia did not impact on motor gains during rehabilitation. While recognising our study was not powered for this subanalysis, the results of our study suggest cognition may impact on physical rehabilitation. The impact was small, with each 1 point increase on the MMSE increasing the odds of responding to rehabilitation by 7 %, however the MMSE is not sensitive to mild change in cognitive status. Further exploration of the relationship between cognition and rehabilitation outcomes using more sensitive measures of cognition is warranted.

Response to rehabilitation was not associated with baseline ambulation status, frailty, comorbidities or depression. A retrospective study of 556 older people undergoing rehabilitation found while age, depression and co-morbidities were associated with functional gain, in multivariate logistic regression only participation, ability to walk pre-admission and admission following orthopaedic surgery remained predictive [10]. Depression was associated with failure to achieve walking independence following hip fracture [37] and was also associated with smaller changes in FIM in older people admitted for rehabilitation [19]. While there is evidence that frailty increases the odds of incomplete recovery following an acute illness, [8] other studies have found frailty does not impact on response to community based rehabilitation [12, 14, 18]. A systematic review concluded that co-morbidities may be associated with rehabilitation outcomes in people with stroke and hip fracture, [9] however co-morbidities were not predictive of change in FIM scores in older people [11] or change in FIM motor scores in a group of younger people (mean age 57.7 years ± 14.6) undergoing rehabilitation [16]. Furthermore, a large study of older Medicare beneficiaries undergoing rehabilitation in the United States of America found co-morbidity indices did not predict functional outcomes [13]. Conflicting results between studies are likely due to the different variables included in the analyses, differences in tools utilised to measure variables such as frailty and co-morbidity and differences in study population. Our study had a high proportion of people with multiple co-morbidities and 72 % scored either 2 or 3 on the Fried Frailty Index. Clustering of frailty scores may explain the lack of association with rehabilitation response and probably reflects selection bias; older people who are not frail may not require rehabilitation following an acute illness, while those who are very frail may be deemed not suitable for rehabilitation. This also highlights that findings from this study are only generalisable to similar populations of older people.

We found the odds of achieving a meaningful change in gait speed increased with an increase in the number of days of rehabilitation but was not associated with the amount of supervised upright physical activity. Rehabilitation was delivered until the person was discharged from hospital-based rehabilitation. Readiness for discharge from rehabilitation was usually decided by the treating team in conjunction with the patient; generally when rehabilitation goals were achieved, or no further gains were likely to be made. However, discharge from rehabilitation may sometimes occur prematurely or be delayed, which may impact on this finding. The number of days in rehabilitation was low, with a median of 16 days in people who responded compared with 14 days in those who did not respond. People with shorter length of stay may not have had sufficient time to achieve a meaningful change in gait speed.

The lack of association between daily supervised upright activity and odds of having a positive response is in keeping with the primary analysis of the RCT, which found additional physical activity did not lead to improved walking outcomes [24]. Links between exercise dosage and outcomes have been established in other populations, such as people with stroke [20–22] and for fall prevention [23]. These results suggest the link is less clear for older people undergoing hospital-based rehabilitation following an acute illness. Recovery during this relatively short time period may reflect recuperation from illness rather than response to exercise. Physical activity during this period may be important in preventing functional decline [38] and loss of muscle strength [39], but higher doses of physical activity during this short time frame may not provide added benefit. This analysis includes supervised upright activity delivered during both usual care physical therapy sessions, which typically provided around 20–25 minutes of upright activity, and intervention sessions, which typically provided an additional 20 minutes of upright activity to participants in the intervention arm. However, the analysis does not include activity conducted outside these sessions. The intervention focussed on upright mobility activities but did not specifically emphasise gait speed training. Our findings highlight the need to further explore optimal dosage and content of physical activity programs for older people undergoing hospital-based rehabilitation.

### Study limitations

This was a rigorously conducted trial; data was prospectively collected from four wards over two hospital sites by an assessor blinded to interventions. However, this was a secondary analysis thus results must be interpreted with caution. People in this trial were admitted with a large range of medical conditions, therefore this was not included as a variable in the analysis. Other studies have shown that people who are admitted with an orthopaedic diagnosis are more likely to have a positive response to rehabilitation [10]. As this analysis was not the primary focus of the trial we did not collect data on other variables that may be predictive of response to rehabilitation, such as participation [37] and change in status over the first one to two weeks [40].

## Conclusions

Approximately two out of every three older people admitted for hospital-based rehabilitation achieved a clinically meaningful improvement in gait speed. Increased days receiving rehabilitation and better cognition, as assessed using the MMSE, were associated with increased odds of having a positive response to rehabilitation. Baseline ambulation, frailty, co-morbidities depression and daily upright activity time were not associated with a positive response to rehabilitation.

## Data Availability

The datasets generated and/or analysed during the current study are not publicly available but are available from the corresponding author on reasonable request.

## Abbreviations

aORs: Adjusted Odds Ratios
CCI: Charlson Co-morbidity Index
CIs: Confidence intervals
FIM: Functional Independence Measure
GDS: Geriatric Depression Scale
IQR: Interquartile range
MMSE: Mini Mental State Examination
ORs: Odds Ratios
ROC: Receiver Operating Characteristics
RCT: Randomized controlled trial
